# Supplementary material for: Adenosine triphosphate is co-secreted with glucagon-like peptide-1 to modulate intestinal enterocytes and afferent neurons
Source: Nat Commun. 2019 Mar 4;10:1029. doi: 10.1038/s41467-019-09045-9 (PMC6399286; doi:10.1038/s41467-019-09045-9)
Supplement: Supplementary file 1 — Supplementary Information [file 41467_2019_9045_MOESM1_ESM.pdf]

Supplementary Information:

Adenosine triphosphate is co-secreted with glucagon-like peptide-1 to modulate intestinal enterocytes and afferent neurons

Lu et al.

Supplementary Figures:

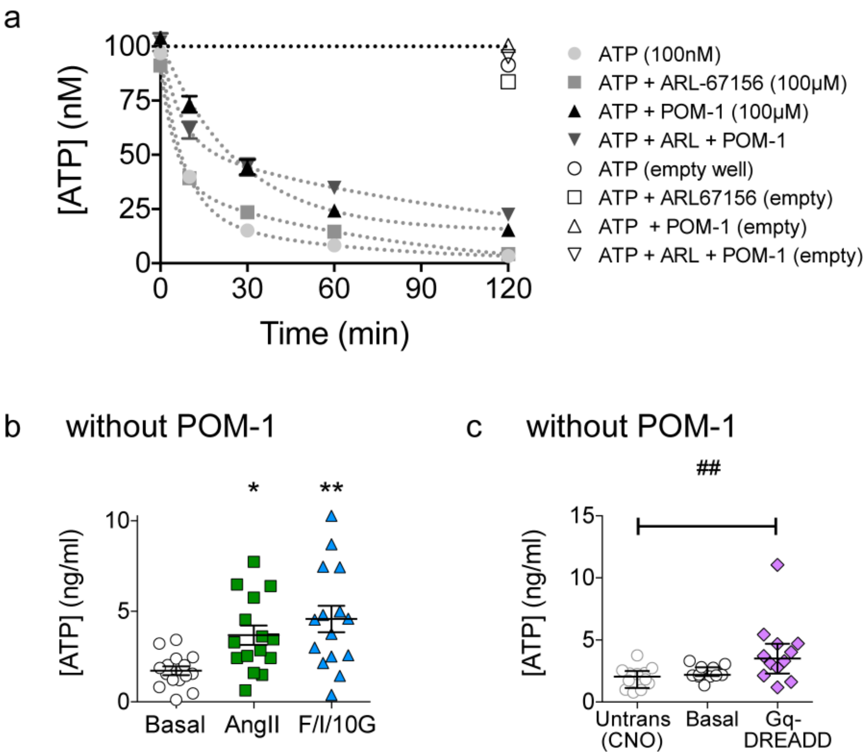

Supplementary Figure 1: Supernatant ATP stability

(a) ATP (100 nM) in standard saline solution was added to plated GLUTag cells (closed symbols) or control wells containing no cells (open symbols) and incubated at 37 °C for the times indicated, with or without ecto-ATPase inhibitors; no inhibitor (circles), ARL-67156 (100 μM, squares), POM-1 (100 μM, upwards pointing triangles), ARL-67156+POM-1 (downward pointing triangles). Dashed lines indicate non-linear curve fits of data to a double exponential function for all groups except a single exponential function for the POM-1 group. Mean ± SEM shown (n = 9 from 3 independent experiments). (b-c) ATP levels measured in GLUTag cell supernatants in the absence of ectonucleotidase inhibition by POM-1. (b) Measurements taken after stimulation for 10 min with AngII (1 μM, green squares) or forskolin (10 μM) + IBMX (10 μM) + 10 mM glucose (F/I/10G, blue triangles); n = 15 wells, data obtained from 5 independent experiments. (c) ATP levels measured in untransfected GLUTag cells treated with CNO (10 μM, Untrans CNO), or GLUTag cells transiently transfected with GqDREADD, incubated without (Basal) or with CNO (10 μM, purple diamonds); n = 12 from 3 independent experiments. Individual points represent measurements from single wells and lines on the graph represent mean ± SEM for (b), or median ± interquartile range for (c). *$p<0.05$, **$p<0.01$ by one-way ANOVA with Dunnett's multiple comparisons test. ##$p<0.01$ by Kruskal-Wallis ANOVA with multiple comparisons test.

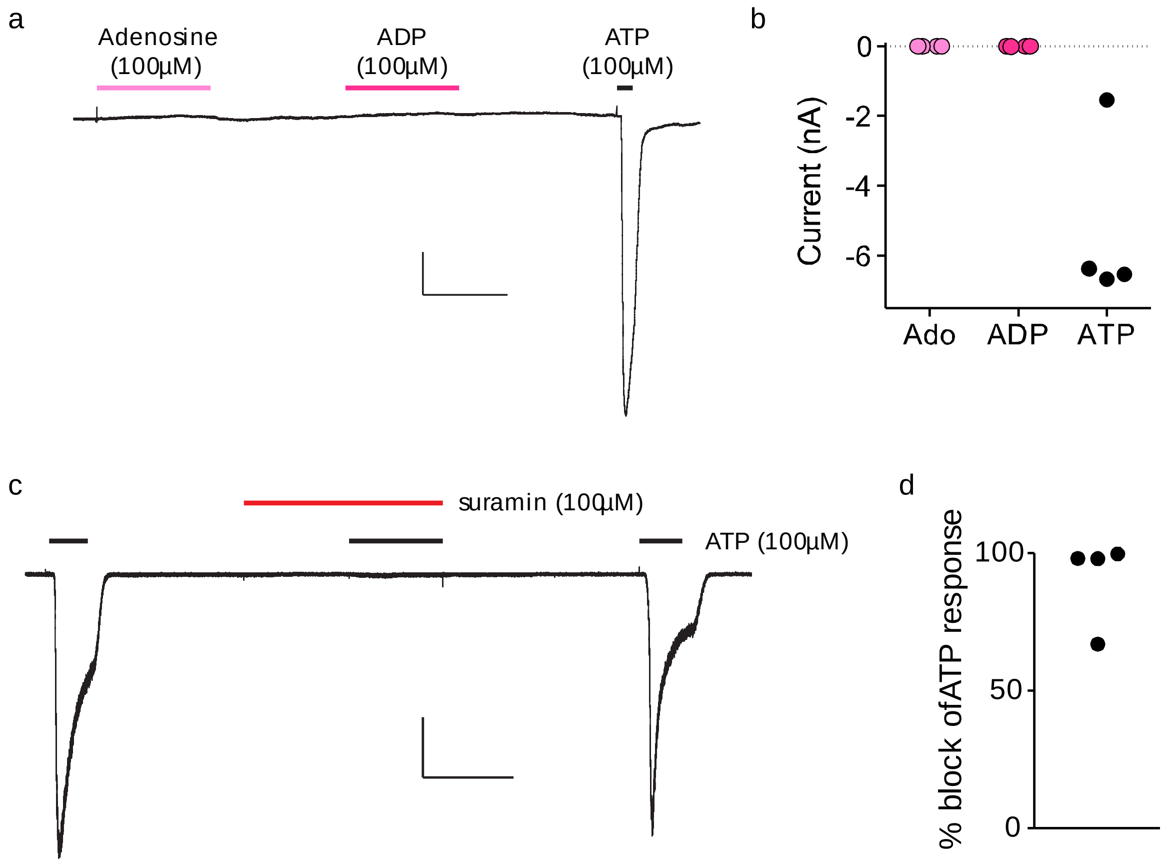

Supplementary Figure 2: Control sniffer patch recordings

(a) Sniffer patch recording from a $P2X_2$-transfected HEK293 cell stimulated with adenosine (100 µM, light pink circles), ADP (100 µM, dark pink circles) and ATP (100 µM, black circles). (b) Summary of $P2X_2$-current amplitudes from sniffer patches. n=4 for all groups. (c) Control sniffer patch recording trace from a $P2X_2$-transfected cell testing the block of ATP-evoked currents by suramin (100 µM). Y-axis scale bar is 1 nA for both (a) and (b), X-axis scale bar is 200 ms for (a) and 50 ms for (b). (d) Summary of $P2X_2$-current block by suramin. N=4.

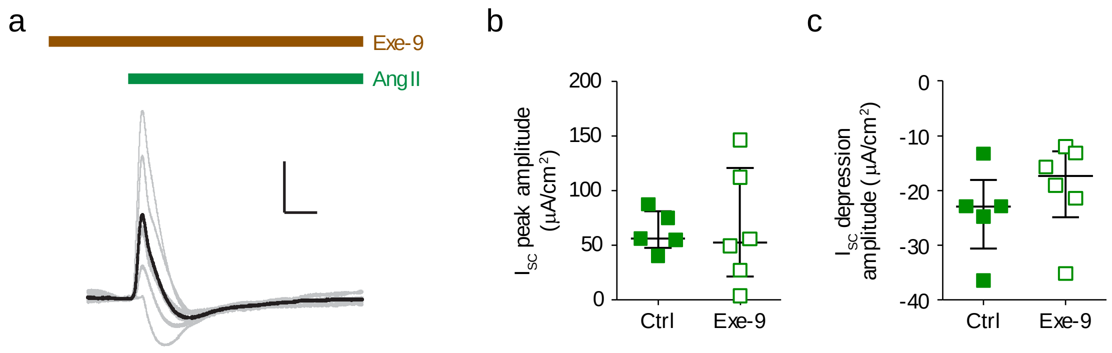

Supplementary Figure 3: Exendin-9 effects on AngII-induced I_{sc} changes

Superimposed short-circuit current (I_{sc}) recordings during basolateral pre-treatment with exendin-9 (Exe-9, 1 µM) and application of angiotensin II (AngII, 1 µM). Application of AngII and Exe-9 (~10 min) indicated above traces. Grey traces indicate individual recordings and black traces represent an average of traces. Y-axis scale bar is 10 µA, X-axis scale bar is 2 min. Scatterplot I_{sc} amplitude of the peak (b) and depression (c) components of I_{sc} responses. Individual data points represent individual epithelial preparations and lines represent median ± interquartile range (n = 5 for control, n = 6 for exendin-9 pre-treated). Statistical analysis performed using Mann-Whitney test, p = 0.77 for (b) and p = 0.18 for (c).

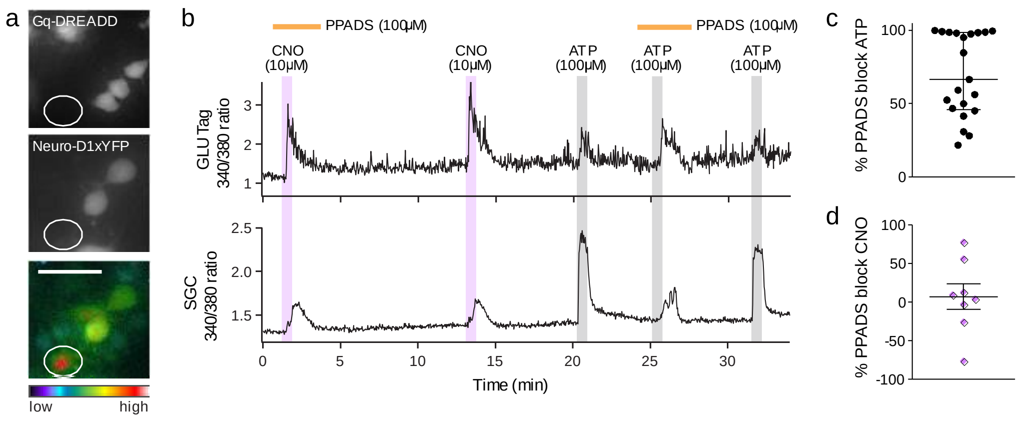

Supplementary Figure 4: Fura-2 Ca^{2+} imaging of satellite glial cells (SGCs)

(a) Fluorescence images of co-culture system at 550 nm excitation (top panel), at 488 nm excitation (middle panel), and the ratio of 340 to 380 nm (lower panel, pseudocoloured 340/380 ratio, scale indicated). Circle indicates an RFP-negative, YFP-negative cell with a robust elevation of Ca^{2+} in response to ATP (100 µM). Scale bar is 50 µm. (b) Intracellular Ca^{2+} levels, represented as the ratio of Fura-2 fluorescence at 340 nm to 380 nm, of an mCherry-positive Gq-DREADD transfected GLUTag cell (top trace) and SGC cell (lower trace). Application of drugs indicated above traces. (c) Scatterplot of the % block of ATP responses in SGCs by 100 µM PPADS. Individual data points represent each SGC cell and lines represent median ± interquartile range (n = 21). (d) Scatterplot of the % block of CNO responses in SGC cells by 100 µM PPADs. Individual data points represent each SGCs and lines represent mean ± SEM (n = 8). Statistical analysis performed using one-sample t-test, p = 0.68.

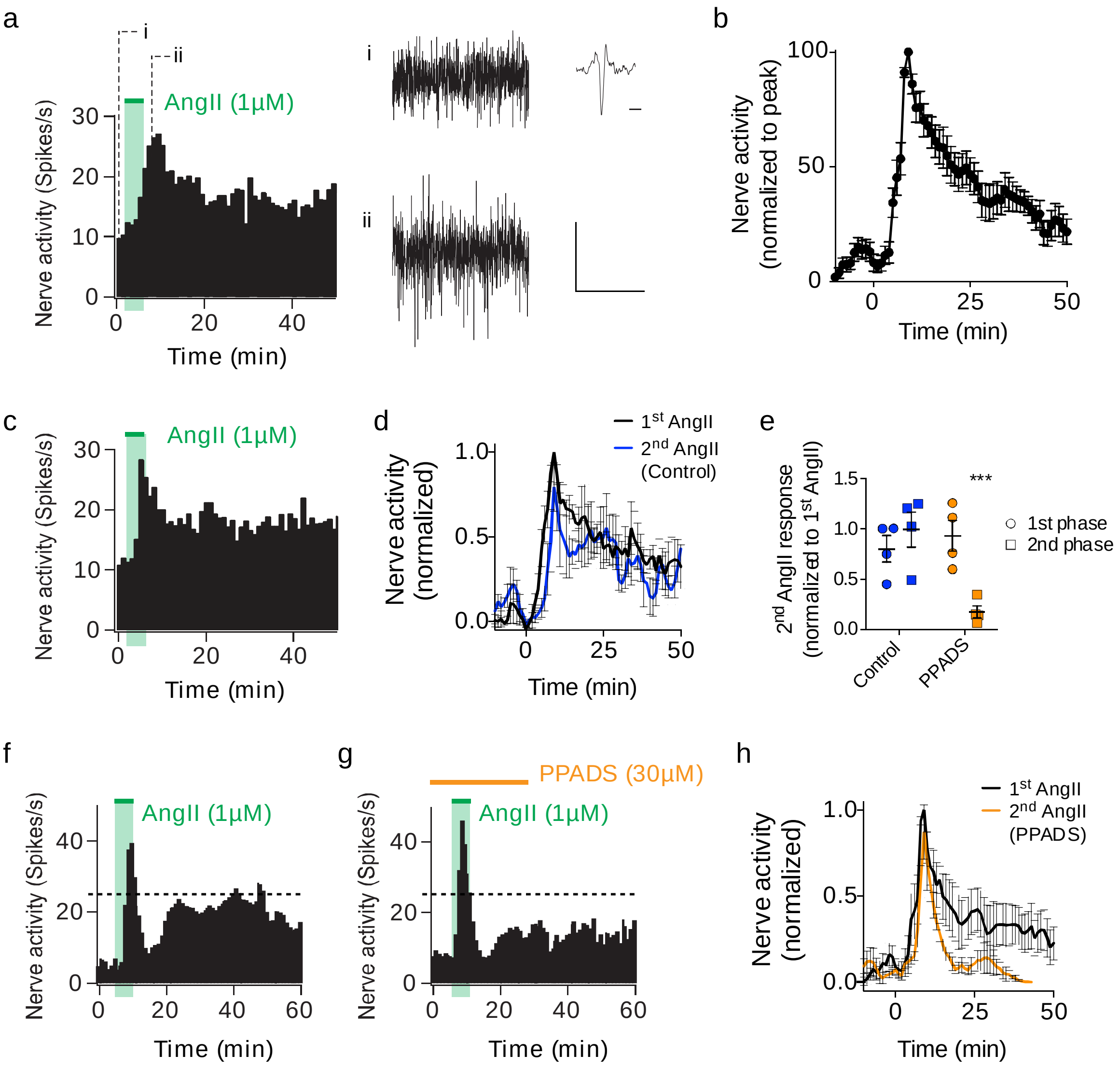

Supplementary Figure 5: Afferent nerve activity in ex vivo proximal colon

(a) Example firing rate histograms in response to angiotensin II (AngII, 1 µM, green bars). i and ii: expanded traces at baseline indicated in a). Y-axis scale bar is 0.1 V, x-axis scale bar is 5 s. A representative spike is shown beside i, with an x-axis scale bar of 2 ms. (b) Response profiles to AngII (n=11). Time = 0 indicates the start of AngII treatment. (c) Firing rate histogram from the same example as in a) in response to a second AngII application (1 µM, green bar). (d) Response profiles to the first AngII application (black line) and second AngII application (blue line)(n=4). (e) Comparison of 1st and 2nd AngII responses (circles and squares, respectively) and effect of PPADS (orange symbols) on 2nd AngII responses. Values for the first phase taken at the peak of the firing rate histogram, which occurred during or within 10 min of AngII application, the second phase taken from the firing rate histogram 15 min after the peak response. Mean ± SEM superimposed on individual data points (n=4). Statistical analysis performed using a one-sample t-test, *** $p<0.001$. Example firing rate histogram for the same colonic preparation in response to AngII

(f) and AngII following pre-treatment with 30 μM PPADS (g) indicated by an orange bar. (h) Response profiles to the first AngII application (black line) and second AngII application in the presence of 30 μM PPADS (orange line)(n=4). All response profiles (b, d, h) baseline subtracted and normalized to the initial peak of the first AngII application, and lines represent mean ± SEM.

Supplementary Tables:

Supplementary Table 1: List of antibodies used

Target	*Primary antibody supplier, catalogue number, host, concentration*	*Secondary antibody, supplier, catalogue number, host, concentration*
Vesicular Nucleotide Transporter (VNUT)	Millipore, ABN83, guinea pig, 1:500	Anti-guinea pig, Jackson ImmunoResearch, 706-545-148, donkey, 1:300
Glucagon-like peptide-1 (GLP-1)	Abcam, ab22625, rabbit, 1:200	Anti-rabbit, Molecular Probes, A31572, donkey, 1:300
Peptide YY (PYY)	Abcam, ab22663, rabbit, 1:200	Anti-rabbit, Molecular Probes, A31572, donkey, 1:300
$P2X_3$	Alomone, APR-016, rabbit, 1:200	Anti-rabbit, Molecular Probes, A31572, donkey, 1:300
$P2X_3$	Neuromics, GP10108, guinea pig, 1:200	Anti-guinea pig, Molecular Probes, A21435, goat, 1:300
GFP	Abcam, ab13970, chicken, 1:2000	Anti-chicken, Jackson ImmunoResearch, 703-545-155, donkey, 1:300 or<br><br>Anti-chicken, Molecular Probes, A11073, goat, 1:300
GLP1-R	MedImmune, humanized mouse, 0.1 mg ml^{-1}	Anti-human, ThermoFisher, SA5-10129, donkey, 1:300 or<br><br>Anti-human, Molecular Probes, A21445, goat, 1:300

Supplementary Table 2: List of primers and TaqMan Assays used

Gene	Forward primer sequence (5' – 3')	Reverse primer sequence (5' – 3')	Assay ID (Applied Biosystems)
Neuro-D-Cre	ATTCCATTTCCAAACTCATCGT	GGACAGAAGCATTTTCCAGGTA	
GLP1R-Cre	CAGCGCCGAACATCTCCTGG	GTAGTCCCTCACATCCTCAGG	
GLU-Cre	GGCATTGGAGCCATAAGCAGG	GTAGTCCCTCACATCCTCAGG	
EYFP	GACGTAAACGGCCACAAGTT	GGATCTTGAAGTTCGCCTTG	
tdRFP	CACCATCGTGGAACAGTACG	CCCATGGTCTTCTTCTGCAT	
GCaMP3	CACATGAAGCAGCACGACTT	AGCCATTGCCATCCTTATCA	
Gq-DREADD	CGCCACCATGTACCCATAC	GTGGTACCGTCTGGAGAGGA	
P2rx1			Mm_00435460_m1
P2rx2			Mm_00462952_m1
P2rx3			Mm_00523699_m1
P2rx4			Mm_00501787_m1
P2rx5			Mm_00473677_m1
P2rx6			Mm_00440591_m1
P2rx7			Mm_01199500_m1
Glp1r			Mm_00445292_m1
Gapdh			Mm99999915_g1
