# Supplementary material for: Adenosine triphosphate is co-secreted with glucagon-like peptide-1 to modulate intestinal enterocytes and afferent neurons
Source: Nat Commun. 2019 Mar 4;10:1029. doi: 10.1038/s41467-019-09045-9 (PMC6399286; doi:10.1038/s41467-019-09045-9)
Supplement: Supplementary file 3 — Description of Additional Supplementary Files [file 41467_2019_9045_MOESM3_ESM.pdf]

# Description of Additional Supplementary Files

File Name: Supplementary Movie 1

Description: Quinacrine fluorescence increase and dissipation in GLUTag cells. Quinacrine dihydrochloride (5 µM, 20 min) was added to plated GLUTag cells and imaged for quinacrine fluorescence using total internal reflection fluorescence microscopy every 50 ms (20 Hz). Shown is an example of a sharp increase in fluorescence, followed by the fluorescence outwardly dissipating, as shown in Figure 1g.
