# Supplementary material for: Adenosine triphosphate is co-secreted with glucagon-like peptide-1 to modulate intestinal enterocytes and afferent neurons
Source: Nat Commun. 2019 Mar 4;10:1029. doi: 10.1038/s41467-019-09045-9 (PMC6399286; doi:10.1038/s41467-019-09045-9)
Supplement: Supplementary file 5 — Reporting Summary [file 41467_2019_9045_MOESM5_ESM.pdf]

nature research

Corresponding author(s): Frank Reimann

Last updated by author(s): Jan 17, 2019

Reporting Summary

Nature Research wishes to improve the reproducibility of the work that we publish. This form provides structure for consistency and transparency in reporting. For further information on Nature Research policies, see Authors & Referees and the Editorial Policy Checklist.

Statistics

For all statistical analyses, confirm that the following items are present in the figure legend, table legend, main text, or Methods section.

n/a	Confirmed	
☐	☒	The exact sample size (n) for each experimental group/condition, given as a discrete number and unit of measurement
☐	☒	A statement on whether measurements were taken from distinct samples or whether the same sample was measured repeatedly
☐	☒	The statistical test(s) used AND whether they are one- or two-sided<br>*Only common tests should be described solely by name; describe more complex techniques in the Methods section.*
☒	☐	A description of all covariates tested
☐	☒	A description of any assumptions or corrections, such as tests of normality and adjustment for multiple comparisons
☐	☒	A full description of the statistical parameters including central tendency (e.g. means) or other basic estimates (e.g. regression coefficient) AND variation (e.g. standard deviation) or associated estimates of uncertainty (e.g. confidence intervals)
☐	☒	For null hypothesis testing, the test statistic (e.g. F, t, r) with confidence intervals, effect sizes, degrees of freedom and P value noted<br>*Give P values as exact values whenever suitable.*
☒	☐	For Bayesian analysis, information on the choice of priors and Markov chain Monte Carlo settings
☒	☐	For hierarchical and complex designs, identification of the appropriate level for tests and full reporting of outcomes
☒	☐	Estimates of effect sizes (e.g. Cohen's d, Pearson's r), indicating how they were calculated

Our web collection on statistics for biologists contains articles on many of the points above.

Software and code

Policy information about availability of computer code

Data collection	Not applicable
Data analysis	The code used for co-localisation and TIRF analysis is available at https://bitbucket.org/cwissmiff/travis/src under an Academic Free License (v3.0).

For manuscripts utilizing custom algorithms or software that are central to the research but not yet described in published literature, software must be made available to editors/reviewers. We strongly encourage code deposition in a community repository (e.g. GitHub). See the Nature Research guidelines for submitting code & software for further information.

Data

Policy information about availability of data

All manuscripts must include a data availability statement. This statement should provide the following information, where applicable:

- Accession codes, unique identifiers, or web links for publicly available datasets
- A list of figures that have associated raw data
- A description of any restrictions on data availability

The source data underlying Figures 1-6 and Supplementary Figures 1-5 are provided as a supplementary information Source Data file. All original raw data files are available from the corresponding author upon reasonable request. Unique biological materials (e.g. transgenic mice) are available for collaborations from the authors upon reasonable request. The code used for co-localisation and TIRF analysis is available at https://bitbucket.org/cwissmiff/travis/src under an Academic Free License (v3.0).

Field-specific reporting

Please select the one below that is the best fit for your research. If you are not sure, read the appropriate sections before making your selection.

☒ Life sciences ☐ Behavioural & social sciences ☐ Ecological, evolutionary & environmental sciences

For a reference copy of the document with all sections, see nature.com/documents/nr-reporting-summary-flat.pdf

Life sciences study design

All studies must disclose on these points even when the disclosure is negative.

Sample size	Sample size was determined by previous experience with the employed preparations. No pro-forma power calculations were perfomed.
Data exclusions	In most cases we did not exclude any data - however, in some cases only data was included in which a "second" response was actually observable. This is clearly explained in the manuscript.
Replication	All experiments were perfomed on several independent preparations. In addition, some key experiments (e.g. ATP accumulation in supernatants, Calcium responses in co-cultures) were performed independently by more than one author, however, this was not possible for some of the more specialised experiments (e.g. sniffer patches, TIRF imaging).
Randomization	Not really applicable to the data presented here.
Blinding	In most cases no blinding was attempted, as this does not easily lend itself to the kind of experiments described in this manuscript. Hormone (GLP-1) secretion includes a blinding as the levels are measured by a service lab blind to the underlying experiment.

Reporting for specific materials, systems and methods

We require information from authors about some types of materials, experimental systems and methods used in many studies. Here, indicate whether each material, system or method listed is relevant to your study. If you are not sure if a list item applies to your research, read the appropriate section before selecting a response.

Materials & experimental systems

n/a	Involved in the study	
☐	☒	Antibodies
☐	☒	Eukaryotic cell lines
☒	☐	Palaeontology
☐	☒	Animals and other organisms
☐	☒	Human research participants
☒	☐	Clinical data

Methods

n/a	Involved in the study	
☒	☐	ChIP-seq
☒	☐	Flow cytometry
☒	☐	MRI-based neuroimaging

Antibodies

Antibodies used	Information is included in Supplementary Table 1.
Validation	We did not further validate the commercially available antibodies. The GLP1R antibody used was raised in collaboration with MedImmune and was validated in house as described in PMID: 29119245

Eukaryotic cell lines

Policy information about cell lines

Cell line source(s)	As stated in the manuscript; GLUTag cells received ~ in 2001 from Dan Drucker (Toronto) and since provided to many other laboratories once MTAs with the Drucker lab are signed; HEK293A cells from Q-BIO-gene.
Authentication	HEK293A cells have been used by Frank Reimann to produce adenoviral particles, so clearly contain adenoviral genome and are only used here as expression platform for P2X2 channels. GLUTag cells have been used in our laboratory for nearly 20 years and secrete GLP-1 (e.g. shown in this manuscript) and have also been verified by RNAseq analysis in our laboratory previously.
Mycoplasma contamination	Mycoplasma testing is performed regularly in MRL - no positive findings.
Commonly misidentified lines (See ICLAC register)	Not applicable

Animals and other organisms

Policy information about studies involving animals; ARRIVE guidelines recommended for reporting animal research

Laboratory animals	Mice, both sexes, C57Bl6-background (mostly C57Bl6J, however, some C57Bl6N contamination, based on breeding /back-crossing for >2years. In this study we used in house generated GLU-Cre12 mice (PMID:22638549) crossed with Rosa26-reporter mice (GCaMP3: JAX:B6;129S-Gt(ROSA)26Sortm38(CAG-GCaMP3)Hze/J, however,backcrossed for >8 generations into C57Bl6JN; Gq-DREADD: JAX:B6N;129-Tg(CAG-CHRM3*,-mCitrine)1Ute/J, however, backcrossed for >8 generations into C57Bl6JN; tdRFP PMID: 17171761 - backcrossed for >8 generations into C57Bl6JN). We also used in house generated GLU-Venus mice (PMID:19041768) and NeuroD1-Cre mice generated by Andy Leiter (PMID:22964416) crossed with an EYFP reporter (B6.129X1-Gt(ROSA)26Sortm1(EYFP)Cos/J), all backcrossed for at least 8 generations into C57Bl6JN.
Wild animals	Not applicable
Field-collected samples	Not appliable
Ethics oversight	All animal procedures were approved by the University of Cambridge Animal Welfare and Ethical Review Body and conformed to the Animals (Scientific Procedures) Act 1986 Amendment Regulations (SI 2012/3039). The work was performed under the UK Home Office Project License 70/7824.

Note that full information on the approval of the study protocol must also be provided in the manuscript.

Human research participants

Policy information about studies involving human research participants

Population characteristics	Not applicable - only tissue derived from consented donors used.
Recruitment	Patients undergoing OP for other reasons were approached by qualified staff at Addenbrooke's hospital Cambridge.
Ethics oversight	All human research in this study was conducted in accordance with the principles of the Declaration of Helsinki and after approval by Cambridge University Hospitals R&D and the Cambridge Central Research Ethics Committee (Ref: 09/H0308/24). All participants gave written consent.

Note that full information on the approval of the study protocol must also be provided in the manuscript.
